# Interpreting Patterns of Gene Expression: Signatures of Coregulation, the Data Processing Inequality, and Triplet Motifs

**DOI:** 10.1371/journal.pone.0031969

**Published:** 2012-02-29

**Authors:** Wai Lim Ku, Geet Duggal, Yuan Li, Michelle Girvan, Edward Ott

**Affiliations:** 1 Department of Physics and the Institute for Physical Science and Technology, University of Maryland, College Park, Maryland, United States of America; 2 Center for Bioinformatics and Computational Biology, Institute for Advance Computer Studies, University of Maryland, College Park, Maryland, United States of America; 3 Department of Computer Science, University of Maryland, College Park, Maryland, United States of America; 4 Department of Cell Biology and Molecular Genetics, University of Maryland, College Park, Maryland, United States of America; University of Turin, Italy

## Abstract

Various methods of reconstructing transcriptional regulatory networks infer transcriptional regulatory interactions (TRIs) between strongly coexpressed gene pairs (as determined from microarray experiments measuring mRNA levels). Alternatively, however, the coexpression of two genes might imply that they are coregulated by one or more transcription factors (TFs), and do not necessarily share a direct regulatory interaction. We explore whether and under what circumstances gene pairs with a high degree of coexpression are more likely to indicate TRIs, coregulation or both. Here we use established TRIs in combination with microarray expression data from both *Escherichia coli* (a prokaryote) and *Saccharomyces cerevisiae* (a eukaryote) to assess the accuracy of predictions of coregulated gene pairs and TRIs from coexpressed gene pairs. We find that coexpressed gene pairs are more likely to indicate coregulation than TRIs for *Saccharomyces cerevisiae*, but the incidence of TRIs in highly coexpressed gene pairs is higher for *Escherichia coli*. The data processing inequality (DPI) has previously been applied for the inference of TRIs. We consider the case where a transcription factor gene is known to regulate two genes (one of which is a transcription factor gene) that are known not to regulate one another. According to the DPI, the non-interacting gene pairs should have the smallest mutual information among all pairs in the triplets. While this is sometimes the case for *Escherichia coli*, we find that it is almost always not the case for *Saccharomyces cerevisiae*. This brings into question the usefulness of the DPI sometimes employed to infer TRIs from expression data. Finally, we observe that when a TF gene is known to regulate two other genes, it is rarely the case that one regulatory interaction is positively correlated and the other interaction is negatively correlated. Typically both are either positively or negatively correlated.

## Introduction

If two genes share a transcriptional regulatory interaction (TRI), one or both of them must be a transcription factor gene (TF gene) which can produce a protein called a transcription factor (TF) that regulates the mRNA expression of the other gene. The collection of genes and TRIs work as a dynamic network enabling cells to function and cope with changes in their environment [1]. The increased availability of high-throughput gene expression data has led to a variety of approaches for inferring TRIs [2]–[6]. A typical assumption of these approaches is that strongly correlated mRNA expression profiles (coexpressed profiles) indicate TRIs between two genes if one or both genes is a TF gene. More sophisticated methods of inferring TRIs integrate gene expression with other information, e.g. position weight matrices from sequence motif analysis, as in [7]. Here, we study the use of gene expression alone in determining TRIs. In particular, we focus on the z-score metric used in the CLR algorithm (described in the Methods section). This metric has been argued to give good performance in inferring TRIs [2]. On the other hand, it has been shown in the case of *Saccharomyces cerevisiae* that gene pairs with a high degree of positive coexpression according to the Pearson correlation coefficients may indicate coregulation by TFs [8]. This raises the question of how to biologically interpret high levels of coexpression between gene pairs, particularly in the case of non-time-course data. In this study, we use publicly available prokaryotic bacterium *Escherichia coli* (*E. coli*) and eukariotic *Saccharomyces cerevisiae* (yeast) microarray expression data (these data are collected under different experimental conditions) along with established TRIs to evaluate the accuracy of different predicted gene pairs. In particular, we consider gene pairs that are coexpressed above a selected threshold level. By comparing these gene pairs to the TRIs in the established networks, we obtain estimates of the precision and recall for the prediction that these pairs are TRIs and the alternate prediction that these pairs are coregulated. Our goal is to provide researchers with information that will aid them in evaluating the reliability of using coexpression data to predict transcriptional regulatory interactions and/or coregulation.

In addition, we will also study and classify fan-out motifs [1]: subgraphs composed of a TF gene that coregulates two genes that do not interact directly. In some algorithms using coexpressed profile data to infer TRIs, these coregulated gene pairs are identified as TRIs if they have coexpressed profiles and one of the genes is a TF gene. Different approaches have been applied to identify non-interacting gene pairs in triplets of significantly coexpressed genes, where the main motivation has been to lower the false positive rate of inferring TRIs [3], [9]–[12]. In this paper, we compare the performances of two prominent approaches. One approach is based on application of the data processing inequality (DPI) [3], [13]. The DPI is a general result that can be rigorously derived and states that if, gene 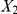 interacts with both genes 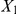 and 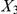 and 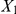 and 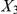 do not interact, then the mutual information between 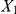 and 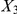 is smaller than the mutual informations of either of the other two gene pairs. More formally, if 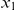, 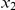, 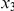 are the expression levels of genes 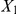, 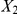, 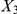, then the DPI is valid if the probability densities for simultaneously observing expression levels 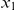 and 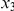 given 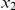 satisfy 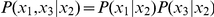. That is, for fixed 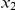, the expression levels 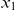 and 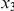 are uncorrelated, and the probability of measuring an expression level 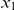 (or 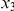) depends only on 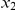 and not on 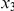 (or 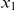). (We emphasize that the satisfaction of this condition of non-interaction of 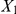 and 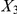 is not clear for actual gene interactions, and we will discuss this subsequently in the Results section.) In contrast to methods assuming applicability of the DPI, another approach claims that the non-interacting gene pairs in fan-out motifs have the maximum mutual information of gene pairs in the triplet [12]. Although [14] points out that application of the DPI in the former approach can fail when mRNA and protein levels of the TF are weakly correlated, this does not necessarily imply the failure of that approach, and the DPI continues to be used by some researchers [3], [13]. One purpose of our study is to address the extent to which the DPI is useful in this context by evaluating its performance using both gene expression and established TRI data. Given these data, we extract fan-out motifs in which at least one of the two non-interacting genes is a TF gene (as is the case when the DPI is commonly applied) and coexpression levels of all gene pairs are above certain thresholds. For each such threshold, we calculate the fraction of the non-interacting gene pairs having the largest, intermediate and smallest mutual information of all pairs in the triplet.

A previous study showed that coregulated gene pairs with a high degree of coexpression tend to be positively correlated [8]. We also explore whether a similar tendency exists in expression correlations between the TF gene and each of the coregulated genes in the datasets we study. In this case, we consider fan-out motifs regardless of whether or not the two coregulated genes interact directly and look for patterns in expression correlations among genes in these three gene subgraphs. To do this, we divide these subgraphs into different types according to the signs of Pearson correlations between gene pairs in the subgraph. There are six such possibilities which we call 
correlation motifs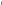. Also, we investigate the classification of these motifs in relation to our obtained mutual information and z-score metrics.

In the following, we first describe the data and the z-score similarity measure. Next, we compare the performance of using coexpression to infer TRIs to that of using coexpression to infer coregulated gene pairs. We then investigate the DPI in fan-out motifs, and we classify these motifs on the basis of the correlations between pairs of genes in the motifs. Conclusions are drawn in the final section.

We emphasize that one of our purposes focuses on testing the validity of the DPI method for pruning indirect interactions, and we have not attempted to test other pruning methods, although our testing techniques could possibly be applied to them. For example, alternative proposed pruning techniques include MRNET [9], conditional mutual information [10], and conditional independence [11]. Also, see Ref. [15] for a comparison of the DPI with some of these methods.

## Methods

### Microarray expression data

We use gene expression microarray data from the Many Microbe Microarray Database (M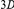) [16] to analyze both *E. coli* and yeast. The expression data consist of a compendium of 445 *E. coli* and 247 yeast Affymetrix Antisense2 microarray expression profiles for 4345 and 5520 genes, respectively. These microarray data were collected under different experimental conditions: different genetic backgrounds, media, growth conditions and perturbing chemicals.

### Known transcriptional regulatory interactions

We use RegulonDB for the established network for *E. coli* and four databases for yeast. We summarize these databases in Table 1.

**Table 1:** The number of TFs, regulated genes and edges in our established TRI data set of known TRIs for *E. coli* and yeast.

Species	Data set of known TRIs	No. of TFs	No. of regulated genes	No. of edges
*E. coli*	RegulonDB	171	1410	3458
yeast	Lee 02A (Chip-chip)	96	2007	3747
yeast	Harbison 04 (Chip-chip/Sequence motif)	99	1732	3186
yeast	Milo 02 (Compilation)	73	550	800
yeast	Lee 02B (Compilation)	87	400	1017

For *E. coli*, we obtain an established network of TRIs from RegulonDB version 6 [17]. 2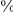 of the genes involving in TRIs from RegulonDB cannot be found in our microarray data. We remove interactions related to those genes from our TRI established network, as well as self-regulatory TRIs. This results in a TRI established network data set consisting of 3458 interactions between 171 TF genes and 1410 genes.

For yeast, a single, generally accepted standard TRI database (analogous to RegulonDB for *E. coli*) has not been established. Therefore, we use four sources of inferred TRIs. As with *E. coli*, we filter out self-regulatory interactions and interactions with genes that are not found in our microarry data.

The **first** database (Lee 02A (Chip-chip)) [18] was obtained using the technology of chromatin immunoprecipitations *in vivo* with microarray (Chip-chip) to identify the binding of TFs to promoter regions in yeast. This database contains 3747 links (bindings) between 96 TFs and 2007 target genes. (Note that the physical bindings of a TF to the promoter regions of a gene does not necessarily imply a regulatory relationship between the TF producing gene and target gene.)

The **second** yeast database (Harbison 04 (Chip-chip/Sequence motif)) [19] was constructed via several steps. First, cis-regulatory sequences, which may act as recognition sites for TFs were identified by combining information from genome-wide location data by Chip-chip, phylogenetically conserved sequences and previously published evidence. Motif discovery methods were applied to these regions in order to discover significant TF-related sequence motifs. Two standards have to be met for these significant motifs in order to conclude the binding of a TF to a promoter region: first, the binding pair is required to have been assigned a high confidence score (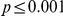) by Chip-chip; second, the promoter sequences are required to be conserved among *sensu stricto Sccharomyces* species. The data set thus obtained includes 3186 interactions between 99 TF genes and 1732 genes.

The **third** yeast database (Milo 02 (Compilation)) [20] was extracted from the Yeast Proteome Database (YPD) [21]. This data set, a compilation from various sources in the literature, provides a list of TRIs including 800 interactions between 73 TF genes and 550 genes and is available to download at www.weizmann.ac.il/mcb/UriAlon.

The **forth** yeast database (Lee 02B (Compilation)) [18] is also a compilation of previously discovered TF-gene bindings (proved by *in vivo* binding, *in vitro* binding, indirect binding and sequence analysis). This collection of interactions is used to compare with the TF-gene binding data from Chip-chip experiments. The result yields 1017 TRIs between 87 TF genes and 400 target genes and can be downloaded at http://web.wi.mit.edu/young/regulator 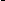
network.

Among our four TRI yeast databases, we believe that the first two (Chip-chip and Chip-chip/Sequence motif) are of generally better quality. We also note that these first two databases (in contrast to the other two) cover almost the whole genome. However, since the four yeast databases may reflect different aspects of the true TRIs, we will give results of analyses using all four.

### Quantifying the similarity of expression profiles

For each pair of genes, we characterize the similarity between their mRNA expression profiles by three metrics: Pearson correlation (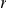), mutual information (MI), and z-score (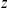). The z-score is used by the CLR algorithm and is related to the empirical distribution of MI values. We here provide a brief review of these metrics.

*The Pearson correlation* 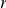. Given 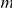 genes (including all TF genes), we compute an estimate of the 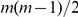 Pearson correlations between gene 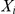 and 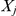, 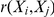, using
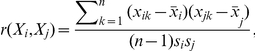
where 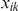
(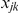) is the gene expression level of gene 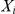
(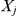) in the 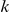
th experimental condition, and 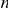 denotes the number of conditions. 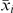
(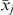) and 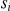
(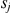) are the mean and standard deviation of the gene expression level of gene 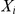
(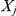).

*The mutual information, MI*. We compute an estimate of the mutual information between genes 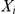 and 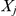 based on the formula,
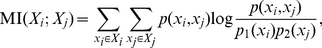
(1)where 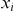
(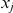) is the variable denoting the expression level of gene 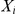
(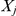). Also, 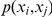 is the joint probability distribution, and 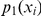 and 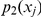 are the marginal probability distribution function for each gene. The expression levels from our databases are continuous variables. To compute the mutual information between continuous random variables, we use a B-spline mutual information estimation code from the M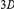 website [16], where this code used a B-spline smoothing and discretization method with 10 bins and third order B-spline to estimate the probabilities in (1) [16], [22].

*The z-score*. The CLR algorithm [2] is an extension of the Relevance network method based on mutual information [3] and uses the z-score between two genes to infer TRIs. The **z-score**, 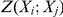, is defined as
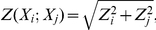
where
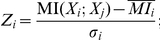


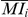
 and 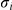 are the mean and standard deviation of the set of values of MI(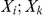), 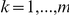.

### Error bars on a fraction

For a sample population of size 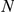, and 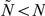 of these measured to have some specific property, the standard error of 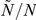 is estimated to be
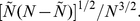
(2)


## Results

As detailed in the Methods section, we obtain microarray expression data for *E. coli* and yeast from M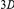 [16], and established transcriptional regulatory interaction data sets from RegulonDB [17] for *E. coli* and from four data sets [18]–[20] for yeast. We use these data in two different types of analyses. In the first type of analysis, we use the z-score metric (described in Methods Section) to determine strongly coexpressed gene pairs, and we compare these with gene pairs in our established TRI data sets. In the second type of analysis, we use the established TRI data together with expression correlation values (using different metrics) to obtain different types of three-gene interaction motifs.

### Signatures of coregulation

There is a question as to whether the degree of coexpression is a predictor of a transcriptional regulatory interaction (TRI), a coregulated gene pair, or both. A high degree of coexpression, as measured by Pearson correlation, has been claimed to indicate coregulated gene pairs [8]. We also note that, a high degree of coexpression between expression profiles of TF-gene pairs, as measured by a high z-score, has been argued to represent TRIs between TF genes and target genes [2]. A benefit of using the z-score to measure the degree of coexpression is that it takes into account the noise in gene expression levels and is therefore considered to be a better measure of coexpression than raw MI. In what follows, we use the z-score to investigate the above question. We find that a high degree of coexpression is more likely to predict coregulated gene pairs for yeast, while it is more likely to predict TRIs for *E. coli*.

**Figure 1:**
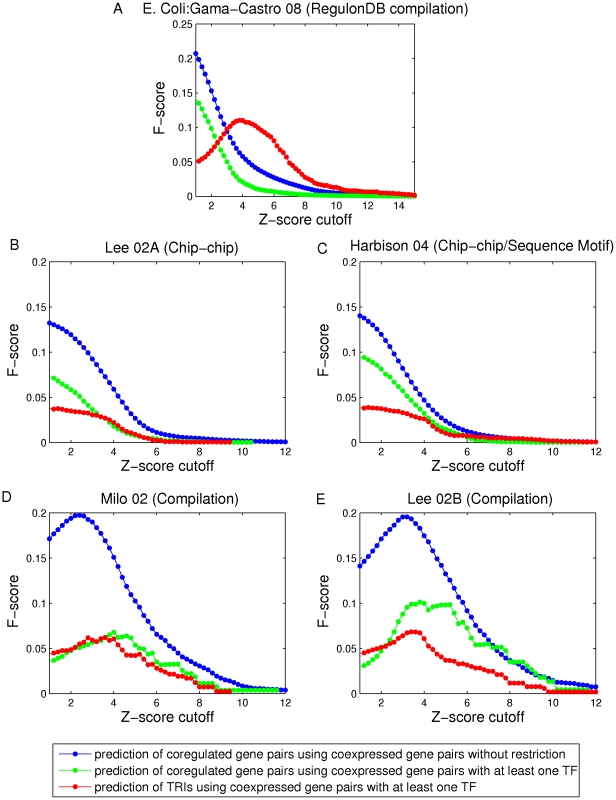
F-score vs. z-score cutoff. F-score versus z-score cutoff for prediction of coregulated gene pairs and TRIs are plotted in blue and red respectively. Also, the F-score curves for the prediction of coregulated gene pairs in coexpression gene pairs with at least one TF gene is plotted in green. The five subplots correspond to the five established TRI data sets for *E. coli* and yeast (Table 1), A) RegulonDB, B) Lee et al. 2002 (Chip-chip), C) Harbison et al. 2004 (Chip-chip/sequence motif), D) Milo et al. 2002 (Compilation) and E) Lee et al. 2002 (Compilation).

When using coexpression to infer TRIs, a TRI is predicted when a gene pair has at least one TF gene and a z-score above a chosen cutoff. When using coexpression to infer coregulation, a gene pair is predicted to be coregulated if its z-score is above a chosen cutoff. To evaluate the quality of these predictions, we use several quantitative measures, namely, the precision (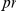), the recall (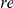), and the F-score. For coregulated gene pairs/TRIs, the precision (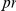) is defined as the ratio of the number of correctly predicted coregulated gene pairs/TRIs to the total number of predicted coregulated gene pairs/TRIs. The recall (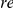) is defined as the ratio of the number of correctly predicted coregulated gene pairs/TRIs to the total number of coregulated gene pairs/TRIs. Then F-score defined as 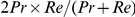, is a measure of the quality of the prediction that reflects the tradeoff between precision and recall. Figure 1 shows plots of F-score versus z-score cutoff for *E. coli* (Fig. 1A) and for yeast (Figs. 1B–E) for three different predictions (the red, green and blue curves). For *E. coli* (Fig. 1A), the F-score for the prediction of coregulated gene pairs (blue curve) is larger than that for TRIs (red curve) when the z-score cutoff is smaller than 3. However, when the z-score cutoff is greater than 3, prediction of TRIs performs better. For the four established TRI data sets of yeast (Figs. 1B–E), F-score values for the prediction of coregulated gene pairs (blue curves) are significantly larger than those for the prediction of TRIs (red curves) for all z-score cutoff, so indicating that the performance of using z-score to predict coregulated gene pairs is better than that of using z-score to predict TRIs. Also, for both predictions of coregulated gene pairs and TRIs (Figs. 1D–E), the plots corresponding to the Milo 02 and Lee 02B TRI data sets have F-score peaks around z-score cutoffs of 3–4 while the other two plots have their maximum F-score at z-score cutoffs of 1. This is an indication for the differences among the TRIs in the four established TRI data sets.

In addition to exploring the incidence of coregulation in all gene pairs with z-score above a certain value, we separately consider only the set of gene pairs with at least one TF gene and z-score above a said value. The corresponding F-score curves are plotted in green in Fig. 1 for both *E. coli* and yeast. For *E. coli*, this green F-score curve is always below that of prediction of coregulated gene pairs from non-restricted coexpressed gene pairs (blue curve). Also, it is below the red F-score curve for prediction of TRIs when z-score cutoff is greater than 2. For yeast, considering Figs. 1B and 1C, we see that the F-score curve for prediction of coregulated gene pairs from restricted coexpressed gene pairs is below that of prediction of coregulated gene pairs from non-restricted coexpressed gene pairs, but above the F-score curve for prediction of TRIs. This indicates that, for both *E. coli* and yeast, coregulated gene pairs with at least one TF are likely to have smaller z-score compared to the unrestricted coregulated gene pairs. We have also studied the precision-recall graphs for all the prediction for both *E. coli* and yeast and the same results are obtained (Shown in Supplementary Figure S1). Our studies reveal that when we go from *E. coli* to yeast, the performance of predicting TRIs using z-score degrades. However, the performance of using z-score to predict coregulated gene pairs from coexpressed gene pairs without restriction is reasonable for both *E. coli* and yeast.

Because the microarray sample size for *E. coli* is much larger than that for yeast, we also employed a sampling approach to demonstrate that the difference in sample sizes does not bias the above conclusions. Specifically, we have recomputed Fig. 1A using randomly selected sets of *E. coli* samples comparable in size to that for our yeast results (Figs. 1B–E). This result, given in the supplementary material (Fig. S2B), shows that the *E. coli* patterns using the smaller sample size are virtually identical to that in Fig. 1A.

Also, TRIs are relatively easier to justify for *E. coli* than for yeast since *E. coli* is a much simpler organism than yeast. This might suggest that the yeast TRI databases are more noisy than the RegulonDB database. In order to demonstrate that noise in yeast TRI databases does not bias our conclusions, we recompute the *E. coli* result (Fig. S2B) with artificially added noise. This was done by randomly deleting 10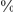 of the links in RegulonDB and then replacing each deleted link by a link from a randomly selected TF gene to a randomly selected gene. This result, given in Fig. S2C of the supplementary material, shows that the *E. coli* patterns in Fig. 1A are robust to adding noise to the TRI database.

The above tests (decrease of the *E. coli* sample size and addition of noise to RegulonDB) confirm the robustness of our conclusion (based on Fig. 1) that when we go from *E. coli* to yeast, the performance of predicting TRIs using z-score degrades while the performance of predicting coregulated gene pairs from coexpressed gene pairs without restriction is reasonable for both *E. coli* and yeast.

### MI-motifs

**Figure 2:**
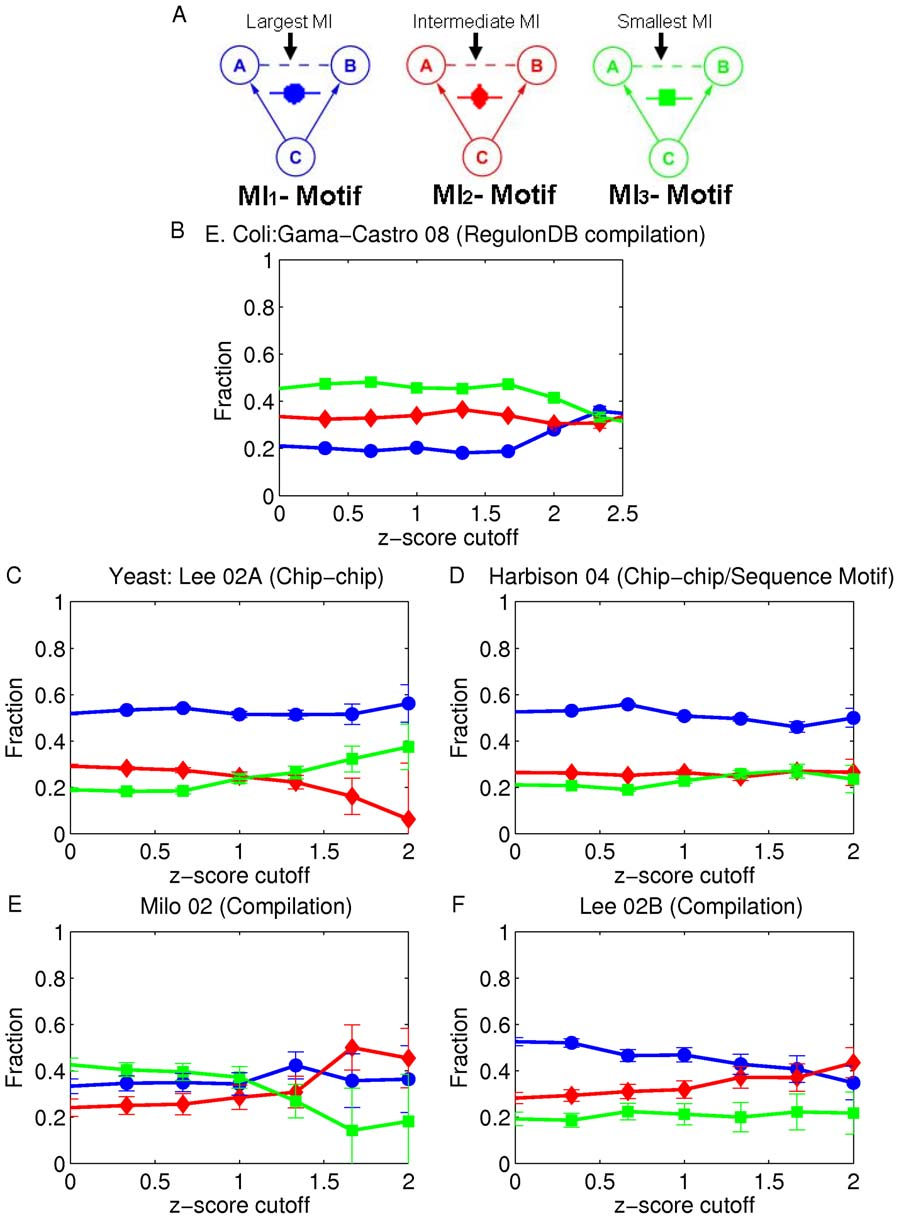
Fractions of MI-motifs vs. the z-score cutoff of non-interacting gene pairs. Non-interacting gene pairs in fan-out motifs are restricted to gene pairs with at least one TF gene. A) MI-motifs in which the non-interacting gene pair has the largest, intermediate and smallest MI. Fractions of MI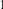, MI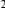 and MI
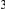
- motifs are plotted in blue, red and green respectively for B) *E. coli* and C–F) yeast. The five subplots correspond to the five established TRI data sets for *E. coli* and yeast (Table 1), B) RegulonDB, C) Lee et al. 2002 (Chip-chip), D) Harbison et al. 2004 (Chip-chip/sequence motif), E) Milo et al. 2002 (Compilation) and F) Lee et al. 2002 (Compilation).

Given an established TRI data set, we can identify all fan-out motifs, where a fan-out motif is defined as a subgraph formed by two non-interacting genes and a TF gene that coregulates them. Here we only consider fan-out motifs in which one of the two coregulated genes is itself a TF gene. The three gene pairs in each fan-out motif are assigned values according to their respective mutual information values. Then we define the three types of MI-motifs shown in Fig. 2A, MI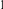, MI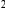 and MI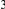, which refer to the case that the value of MI of the non-interacting gene pair is the largest, intermediate and smallest as compared to that of the two TF-gene pairs respectively. If more fan-out motifs are identified as MI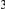-motifs, the data processing inequality(DPI) is a good tool for inferring the non-interacting gene pairs in fan-out motifs. Conversely, if MI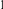-motifs dominate, the non-interacting gene pairs predominantly have the largest MI values within their fan-out motifs, and one might predict that the largest MI indicates coregulation in such a situation, we call this the 
max MI approach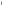 [12].

In order to address the utility of the DPI in this context, we compare the relative abundances of the three MI-motifs in the set of fan-out motifs described above, and we assess how the coexpression levels of gene pairs in fan-out motifs is related to these relative abundances. To do this, we generate different groups of fan-out motifs as we vary the z-score cutoff. For each z-score cutoff, we include only those fan-out motifs in which all gene pairs have a z-score above the cutoff. For each group of fan-out motifs, we compare the relative abundance of the three MI-motifs. We plot the fractions of the three MI-motifs found as a function of the z-score cutoff on all gene pairs. Figs. 2B–F show results for both *E. coli* and yeast. For *E. coli* (Fig. 2B), the relative abundance of MI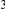-motif is always higher than 40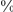 while that of MI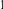-motif is always lower than 25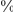. When the z-score cutoff is larger than 2, the relative abundances of MI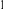, MI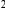 and MI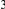-motifs have no distinguishable differences. For the analyses of the Lee 02A, Harbison 04 and Lee 02B data sets of yeast (Figs. 2C, D and F), the relative abundances of MI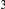-motif are always lower than 30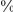 while those of MI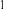-motif are always higher than 40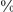. Especially, for the analyses of the Lee 02A and Harbison 04 data sets, the relative abundances of MI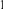-motif are always around 50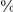. However, for the analysis of the Milo 02 data set (Fig. 2E), the relative abundances of the three MI-motifs are similar and cannot be distinguished. For all four yeast databases, there is no obvious increasing/decreasing trend for these relative abundances with increasing z-score cutoff. This implies that the DPI in the case of *E. coli* works better than the max MI approach and the random prediction for inferring non-interacting gene pairs in fan-out motifs (relative abundance of each MI-motif is equal to one-third in random prediction). However, the performances of the DPI and the max MI approaches are the opposite for yeast. The max MI approach works better than the random case while the DPI fails in inferring non-interacting gene pairs in fan-out motifs. (i.e., the DPI prediction is more often false than a random unweighted guess of the non-interacting links).

Similar to Fig. S2, of the supplementary material, we show in Fig. S3 that the main important features of Fig. 2B are robust to decrease of the *E. coli* sample size to be comparable to the yeast sample size, and also robust to add noise to the *E. coli* TRI database.

In order to demonstrate that our results are not sensitive to the method used for mutual information estimation (a B-spline estimator), we have recomputed Fig. 2B for *E. coli* and Figs. 2C–F for yeast using both empirical [9] and Miller-Madow [23] estimators with both equal-width and equal-frequency binning (10 bins for both). We choose these two estimators because it has been shown that the ARACNE inference method (a method based on DPI) gives the better performance when using these two estimators with equal-frequency binning [15]. The results are given in the supplementary material (Figs. S4, S5, S6, S7, S8), and show that both the *E. coli* and yeast results recomputed using the empirical and Miller-Madow mutual information estimators with both equal-width and equal-frequency are similar to those in Fig. 2B and Figs. 2C–F. In particular as before, for *E. coli* the DPI approach for pruning the non-interacting links in fan-out motifs works better than random and the max MI approach, but it works worse than random in yeast in general.

Regarding the strikingly poor performance in yeast, we note that the DPI, while a rigorous result, only applies when the hypothesis under which it was derived applies (see Introduction Section), and it is unclear to what expect this is the case for gene expression data. One mechanism violating the necessary hypothesis is the possible imperfect correlation between a TF's mRNA level and the production rate of its protein (see Ref. [14]). Another mechanism that would have an equivalent effect is that it can take considerable time for mRNA to be translated into its protein, and thus there can be a significant time lag between the expression levels of a TF and that of its target genes. Still another mechanism that might be relevant is that the expression of target genes may be dependent, not only on the presence of the TF protein involved in the fan-out motif considered, but may also be strongly influenced by other fluctuating factors. Our results suggest that at least one mechanism like those above is most often operative in yeast, but not in *E. coli*. Therefore, the applicability of the data processing inequality may be organism-dependent.

### Correlation-motifs

**Figure 3:**
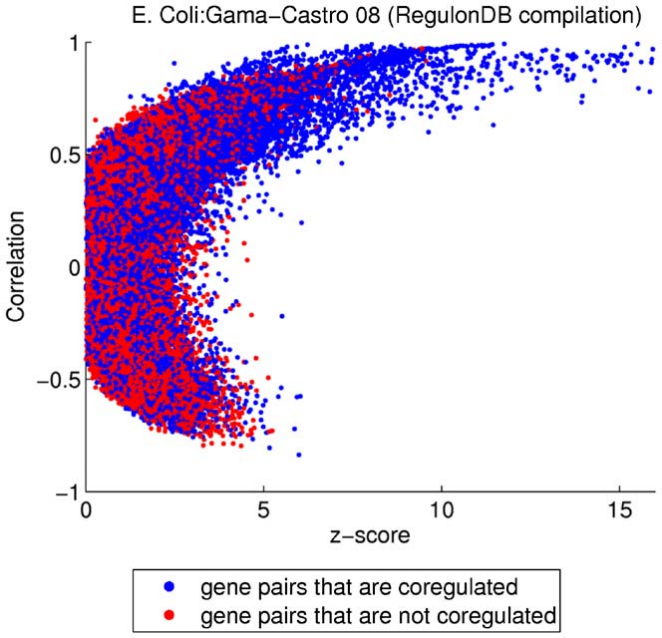
Pearson correlation vs. z-score. Gene pairs that are coregulated are represented by blue dots and those that are not coregulated are represented by red dots for *E. coli*.

A previous study showed that coregulated gene pairs with a large magnitude of Pearson correlation coefficient between their expression profiles tend to be positively correlated [8], [24]. In our study, instead of using Pearson correlation, we will use the z-score metric to measure the degree of coexpression. An initial question is whether the previously found pattern in expression correlation of coregulated gene pairs [8], [24] also appears when the z-score metric is used to quantify coexpression. Figure 3 shows a plot of Pearson correlation versus z-score for *E. coli*. In this figure, gene pairs that are coregulated and not coregulated according to RegulonDB compilation are plotted as blue and red dots respectively (plots for yeast turn out to show similar features to the plot for *E. coli* and are not shown here). To meaningfully represent relative densities of coregulated (blue) and not coregulated (red) pairs in the presence of overlapping of the printed points, we plot points one by one, alternating between blue and red and selecting the gene pairs in the chosen group (blue and red) randomly. This plot shows that a high z-score (z-score 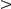
6) is associated with positive correlation and that high z-score gene pairs are likely to be coregulated [the density of blue dots (coregulated gene pairs) is higher than that of red dots (gene pairs that are not coregulated) when the z-score is high]. Motivated by this finding, we consider the situation when a TF gene regulates two other genes, and we ask whether other patterns exist in expression correlation between the TF gene and each of the coregulated genes when coregulated gene pairs have a high degree of coexpression.

**Figure 4:**
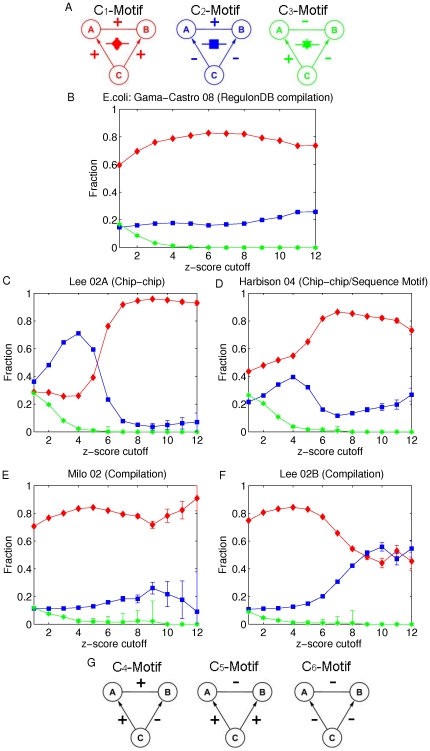
Fractions of C-motifs in a group of subgraphs of coregulation vs. z-score cutoff on coregulated gene pairs in the group. A) C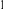, C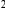 and C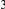-motifs. B–F) The fractions of C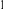, C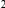 and C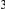-motifs are plotted in red, blue and green respectively. The five subplots correspond to the five established TRI data sets for *E. coli* and yeast (Table 1), B) RegulonDB, C) Lee et al. 2002 (Chip-chip), D) Harbison et al. 2004 (Chip-chip/sequence motif), E) Milo et al. 2002 (Compilation) and F) Lee et al. 2002 (Compilation). G) C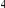, C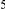 and C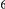-motifs.

We refer to the TF gene and the two genes that it regulates as a coregulation subgraph and we identify these subgraphs from the established TRI databases. However, in contrast to fan-out motifs (discussed in the last section), coregulated genes in these coregulation subgraphs may or may not interact directly. To further explore the correlation and coexpression among genes in coregulation subgraphs, we define six correlation-motifs (C-motifs) by classifying the coregulation subgraphs into different types according to the combinations of the signs of Pearson correlation between the expression of coregulation subgraph genes. There are six such types as shown in Figs. 4A and 4G, where C denotes the TF gene and the other two genes are denoted A and B. The 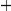 and 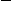 signs on the links denote positive and negative Pearson correlation. We apply Fisher's **z**-transformation to the coefficients of Pearson correlation and obtain the 95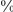 confidence intervals for all coefficients [25]. Among all coregulation subgraphs, we only consider cases where all Pearson correlation coefficients have confidence intervals indicating they have less than a 5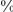 probability to be of the opposite sign.

Next we investigate how the relative abundances of the six C-motifs depends on the z-score between the A and B genes. We first generate different groups of coregulation subgraphs using different z-score cutoffs on the coregulated gene pairs, and for each group, we calculate the relative abundances of the six C-motifs amongst all coregulation subgraphs. Figures 4B–F show plots of the fractions of different C-motifs as a function of the z-score cutoff on coregulated gene pairs for both *E. coli* and yeast. Only the fractions of C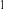, C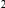 and C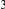-motifs are shown (respectively plotted in red, blue and green) as those of the other C-motifs (Fig. 4G) are very small at all z-score cutoffs. For *E. coli* (Fig. 4B), when the z-score cutoff is above 2, the fractions of C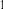 and C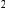-motifs are always about 75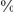 and 18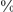 respectively, and the fraction of C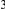-motifs is always lower than those of C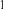 and C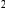-motifs and decreases to near zero around a z-score cutoff of 5. For yeast (Figs. 4C–F), the C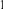 and C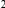-motifs are again the most abundant, while C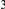-motifs are the least abundant and their fractions decrease to near zero when the z-score cutoffs are high enough (around 6). In particular, for the analysis using the Lee 02A TRI data set (Fig. 4C), C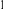-motifs are more abundant than C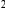-motifs when the z-score cutoff is higher than about 5.5, but they are less abundant than C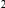-motifs when the z-score cutoff is lower than 5.5. For the analyses using the other three TRI yeast data sets (Figs. 4D, 4E and 4F), C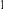-motifs are generally more abundant than C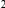-motifs (except for Fig. 4F for the cutoffs greater than 8, where they are approximately equal). The observed differences between the analyses of the four different yeast TRI data sets indicates that there may be significant differences in coregulated genes in different data sets. Overall, results from both *E. coli* and yeast are consistent with our Fig. 3 in that coregulated gene pairs with a high degree of coexpression are more likely to be positively correlated. In addition, these results also imply that when coregulated gene pairs have a large enough z-score, the correlations between the TF gene and the two other genes in the coregulation subgraphs both have the same correlation sign (i.e., they are C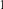 or C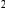 motifs).

**Figure 5:**
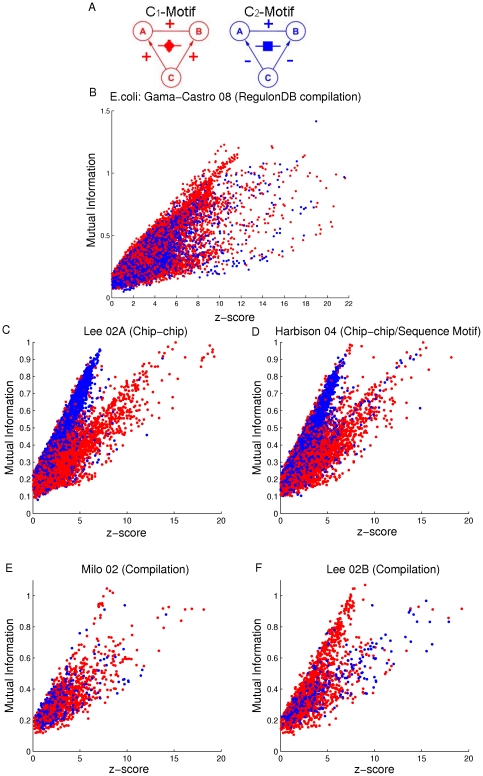
Mutual information vs. z-score for coregulated gene pairs in C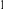 and C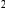-motifs. A) C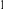 and C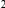-motifs. B–F) Data points for coregulated gene pairs in C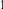 and C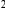-motifs are plotted in red and blue respectively. The five subplots correspond to the five established TRI data sets for *E. coli* and yeast (Table 1), B) RegulonDB, C) Lee et al. 2002 (Chip-chip), D) Harbison et al. 2004 (Chip-chip/sequence motif), E) Milo et al. 2002 (Compilation) and F) Lee et al. 2002 (Compilation).

We now further characterize the difference between the coregulated gene pairs in C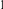 and C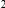-motifs used in the plots of Figs. 4B–F. For each coregulated gene pair, we find their respective mutual information and z-score. Then we construct scatter plots of mutual information versus z-score for all these coregulated gene pairs for both *E. coli* and yeast (Fig. 5) where points corresponding to C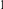-motifs are plotted in red and those corresponding to C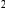 motifs are plotted in blue. There are more C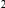-motifs (blue) than C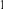-motifs (red). Since overlapping is present, the order in which we plot the points is significant (as for our previous figure, Fig. 3). In the present case we proceed as follows. We first plot randomly selected blue (C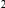-motifs) points until the number of remaining unplotted C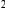-motifs is equal to the number of the C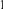-motifs. After that, points are plotted one by one, alternating between randomly selected C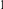-motifs and randomly selected C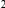-motifs. For *E. coli*, data points for coregulated gene pairs in C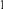-motif are well mixed with those for coregulated gene pairs in C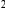-motif in Fig. 5B. Thus there is no apparent distinction observed between coregulated gene pairs in C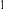 and C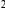-motifs for *E. coli*. Our analyses of the Lee 02A and Harbison 04 yeast data sets (Figs. 5C and 5D) show that mutual information is approximately linearly related to z-score for both groups of coregulated gene pairs (corresponding to blue and red), and that, the slope of the linear relationship for C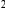-motifs (blue) is larger than that for C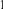-motifs (red). However, distinct slopes are not observed in the analyses of the other two yeast established TRI data sets (Figs. 5E and 5F). We do not presently have a good idea as to a mechanism leading to the observed distinctive 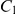 and 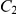 patterns seen in Figs. 5C and 5D.

Regarding a possible reason for the presence of the splitting observed in Figs. 5C and 5D versus the lack of such a splitting in Figs. 5E and 5F, we note that the links in the Milo 02/Lee 02B databases (used for Figs. 5E and 5F) are very different from those in the Lee 02A/Harbison 04 databases (used for Figs. 5C and 5D). In particular, the Lee 02A and Harbison 04 TRI databases are based on Chip-chip experiments, while links in Milo 02 and Lee 02B are inferred by several different methods. It has been shown that different TRI inference methods, such as Chip-chip, targeted gene disruption, and overexpression of TFs, capture distinct facets of the transcriptional regulatory program, and uncover disparate biological phenomena [26]. The fact that a splitting feature is observed in Figs. 5C and D but not in Figs. 5E and 5F may be because different biological processes are reflected in their database constructions.

## Discussion

Our study demonstrates that the performances of prediction of coregulated gene pairs and transcriptional regulatory interactions determined by coexpression levels are organism dependent. For *Escherichia coli*, the prediction of transcriptional regulatory interactions outperforms prediction of coregulated gene pairs when the predictions are determined by coexpression with z-score greater than 3. However, the situation is very different for *Saccharomyces cerevisiae*, with the prediction of coregulated gene pairs outperforming the prediction of TRIs for all z-score cutoffs. Many methods of inferring transcriptional regulatory interactions or coregulated gene pairs have been developed and shown to give excellent performance in specific organisms. However, based on our study, applications of these methods to other organisms should be conducted with caution as their predicting powers may depend on the organism studied.

The Data processing inequality (DPI) has been applied to the prediction of transcriptional regulatory interactions after excluding highly coexpressed gene pairs that do not interact directly. The results show that the application of the DPI to *Escherichia coli* data works better than random prediction of gene pairs. However, the performance of the application of DPI in *Saccharomyces cerevisiae* is worse than that of random prediction. The strong failure of applying DPI to yeast data suggests that factors/mechanisms exist in yeast that lead to an imperfect correlation between the protein and mRNA levels of TFs.

In our study investigating patterns of expression correlation among genes in coregulation subgraphs, we find two distinct types of coregulated gene pairs: one in which the correlation between the expression of the TF gene and both its two target correlated genes are positive and another in which they are both negative. In particular, we present scatter plots of mutual information versus z-score for these two types of gene pairs. The plots for yeast reveal that the two types of coregulated gene pairs split into two parts, thus characterizing the differences between these two types of gene pairs. Further studies are needed to explain the mechanism leading to this behavior.

Motivated by the increasing availability high-throughput gene expression data, a variety of approaches have been developed to infer TRIs or gene coregulation. Our studies in this paper reveal that some approaches which apparently lead to useful prediction in some model organisms may fail in other organisms.

## Supporting Information

Figure S1 **Precision vs. recall.** A–E) Precision versus recall for prediction of coregulated gene pairs and TRIs are plotted in blue and red, respectively. Also, the precision-recall curve for the prediction of coregulated gene pairs in coexpression gene pairs with at least one TF gene is plotted in green. The five subplots correspond to the five established TRI data sets for *E. coli* and yeast (Table 1), A) RegulonDB, B) Lee et al. 2002 (Chip-chip), C) Harbison et al. 2004 (Chip-chip/sequence motif), D) Milo et al. 2002 (Compilation) and E) Lee et al. 2002 (Compilation).
(TIFF)

Figure S2
**F-score vs. z-score cutoff for** ***E. coli*** **.** F-score versus z-score cutoff for prediction of coregulated gene pairs and TRIs are plotted in blue and red, respectively. Also, the F-score curves for the prediction of coregulated gene pairs in coexpression gene pairs with at least one TF gene is plotted in green. A B-spline estimator is used to calculate the mutual information. The three subplots, A, B and C, correspond to different number of samples, A) uses 445 samples (this figure is the same as Fig. 1A in the manuscript), B) uses 194 samples, and C) uses 194 samples and adds noise. The number 194 is derived from 247 (samples for yeast in the data used to derive Figs. 1B–E) 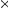 4345 (*E. coli* genes) 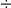 5520 (yeast genes) = 194. For B), the smaller number of samples was obtained by random selecting from the 445 *E. coli* microarray samples used in A). For C), the number of sample is the same as B), and 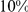 of the links in RegulonDB are deleted and each deleted link is replaced by a link from a randomly selected TF gene to a randomly selected gene. The fact that these figures are virtually identical confirms that any difference between our result in A) with the corresponding yeast results (Figs. 1B–E) is not due to the larger sample size of the *E. coli* microarray database or to lower noise in the RegulonDB database relative to our yeast databases.(TIFF)

Figure S3
**Fractions of MI-motifs vs. the z-score cutoff of non-interacting gene pairs for** ***E. coli*** **.** Non-interacting gene pairs in fan-out motifs are restricted to gene pairs with at least one TF gene. A) MI-motifs in which the non-interacting gene pair has the largest (MI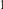 schematic), intermediate (MI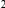 schematic) and smallest (MI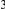 schematic) MI. Fractions of MI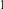, MI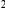 and MI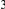 motifs are plotted in blue, red, and green, respectively. A B-spline estimator is used to calculate the mutual information. As in Fig. S2, the three subplots, B, C and D, correspond to B) 445 samples (this is the same as Fig. 2B in the manuscript), C) 194 samples, and D) 194 samples plus noise.(TIFF)

Figure S4
**Fractions of MI-motifs vs. the z-score cutoff of non-interacting gene pairs for** ***E. coli*** **with using different MI estimators as in** **Fig. 2B** **.** A) MI-motifs in which the non-interacting gene pair has the largest, intermediate and smallest MI. Fractions of MI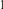, MI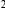 and MI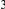 - motifs are plotted in blue, red and green respectively. The five subplots correspond to the use of different MI estimators and discretization methods, B) B-spline (this is the same figure as in Fig. S3C), C) Empirical [9] and equal width (eqw), D) Miller-Madow (MM) [23] and equal width (eqw), E) Empirical and equal frequency (eqf) and F) Miller-Madow (MM) and equal frequency (eqf). These plots show that the conclusion that the green plot is generally above the red and blue plots is independent of the MI estimator that is employed.(TIFF)

Figure S5
**Fractions of MI-motifs vs. the z-score cutoff of non-interacting gene pairs for Lee 02A (Chip-chip) of yeast as in** **Fig. 2C** **.** A) MI-motifs in which the non-interacting gene pair has the largest, intermediate and smallest MI. Fractions of MI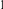, MI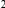 and MI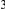 - motifs are plotted in blue, red and green respectively. The five subplots correspond to the use of different MI estimators and discretization methods, B) B-spline (this is the same figure as in Fig. 2C), C) Empirical [9] and equal width (eqw), D) Miller-Madow (MM) [23] and equal width (eqw), E) Empirical and equal frequency (eqf) and F) Miller-Madow (MM) and equal frequency (eqf). These plots show that (in contrast to Fig. S4) the green plot is consistently below the blue plot independent of the MI estimator that is employed.(TIFF)

Figure S6
**Fractions of MI-motifs vs. the z-score cutoff of non-interacting gene pairs for Harbison 04 (Chip-chip/Sequence Motif) of yeast as in** **Fig. 2D** **.** A) MI-motifs in which the non-interacting gene pair has the largest, intermediate and smallest MI. Fractions of MI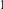, MI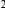 and MI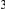 - motifs are plotted in blue, red and green respectively. The five subplots correspond to the use of different MI estimators and discretization methods, B) B-spline (this is the same figure as in Fig. 2D), C) Empirical [9] and equal width (eqw), D) Miller-Madow (MM) [23] and equal width (eqw), E) Empirical and equal frequency (eqf) and F) Miller-Madow (MM) and equal frequency (eqf). These plots show that (in contrast to Fig. S4) the green plot is consistently below the blue plot independent of the MI estimator that is employed.(TIFF)

Figure S7
**Fractions of MI-motifs vs. the z-score cutoff of non-interacting gene pairs for Milo 02 (Compilation) of yeast as in** **Fig. 2E** **.** A) MI-motifs in which the non-interacting gene pair has the largest, intermediate and smallest MI. Fractions of MI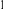, MI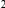 and MI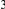 - motifs are plotted in blue, red and green respectively. The five subplots correspond to the use of different MI estimators and discretization methods, B) B-spline (this is the same figure as in Fig. 2E), C) Empirical [9] and equal width (eqw), D) Miller-Madow (MM) [23] and equal width (eqw), E) Empirical and equal frequency (eqf) and F) Miller-Madow (MM) and equal frequency (eqf). These plots show that (in contrast to Fig. S4) the green plot is consistently below the blue plot independent of the MI estimator that is employed.(TIFF)

Figure S8
**Fractions of MI-motifs vs. the z-score cutoff of non-interacting gene pairs for Lee 02B (Compilation) of yeast as in** **Fig. 2F** **.** A) MI-motifs in which the non-interacting gene pair has the largest, intermediate and smallest MI. Fractions of MI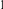, MI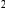, and MI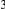 - motifs are plotted in blue, red and green respectively. The five subplots correspond to the use of different MI estimators and discretization methods, B) B-spline (this is the same figure as in Fig. 2F), C) Empirical [9] and equal width (eqw), D) Miller-Madow (MM) [23] and equal width (eqw), E) Empirical and equal frequency (eqf) and F) Miller-Madow (MM) and equal frequency (eqf). These plots show that (in contrast to Fig. S4) the green plot is consistently below the blue plot independent of the MI estimator that is employed.(TIFF)
