# Supplementary figures and images for: Interpreting Patterns of Gene Expression: Signatures of Coregulation, the Data Processing Inequality, and Triplet Motifs
Source: PLoS One. 2012 Feb 29;7(2):e31969. doi: 10.1371/journal.pone.0031969 (PMC3290541; doi:10.1371/journal.pone.0031969)

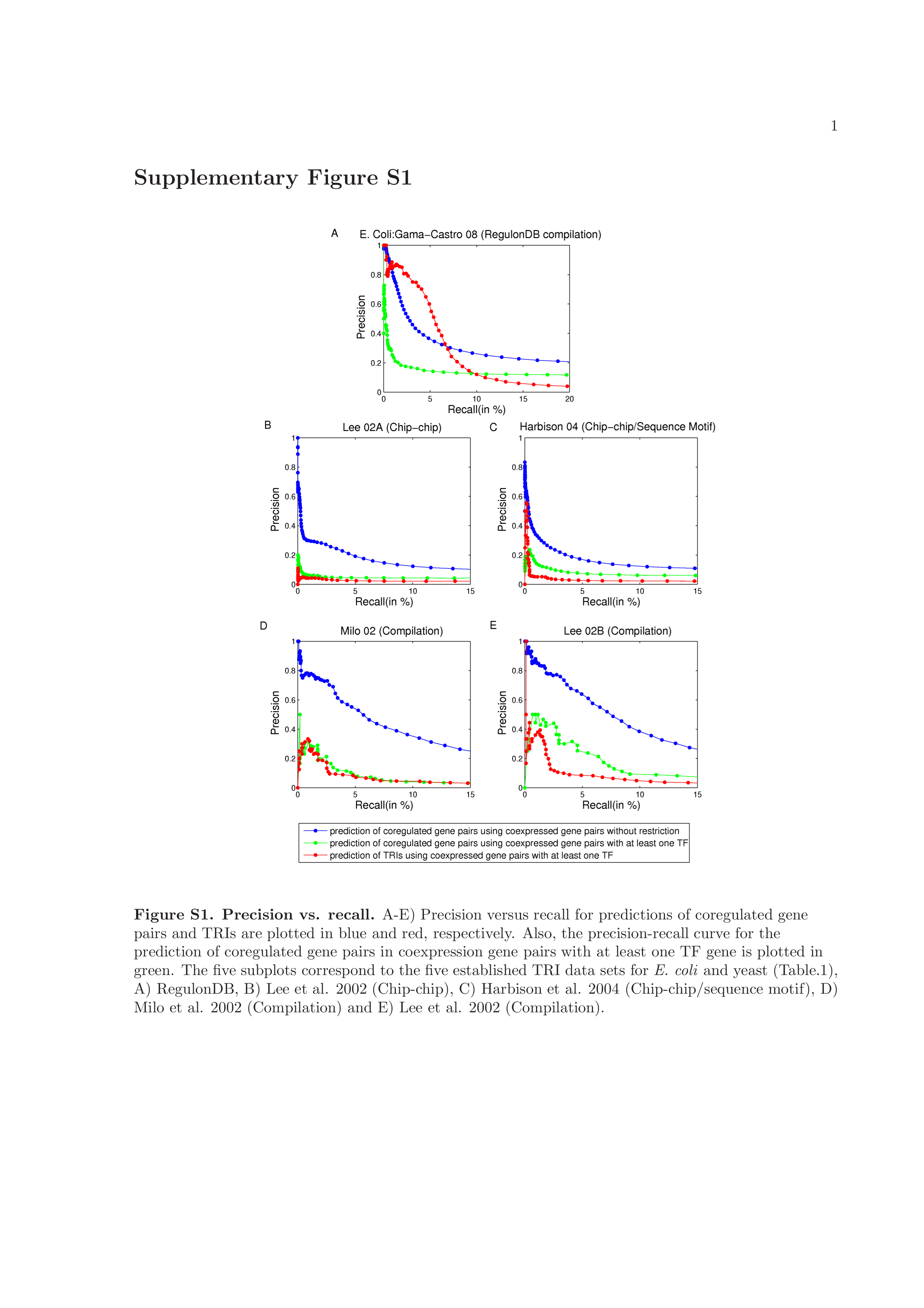

Supplement: Figure S1 — Precision vs. recall. A–E) Precision versus recall for prediction of coregulated gene pairs and TRIs are plotted in blue and red, respectively. Also, the precision-recall curve for the prediction of coregulated gene pairs in coexpression gene pairs with at least one TF gene is plotted in green. The five subplots correspond to the five established TRI data sets for E. coli and yeast (Table 1), A) RegulonDB, B) Lee et al. 2002 (Chip-chip), C) Harbison et al. 2004 (Chip-chip/sequence motif), D) Milo et al. 2002 (Compilation) and E) Lee et al. 2002 (Compilation). (TIFF) [file pone.0031969.s001.tif]

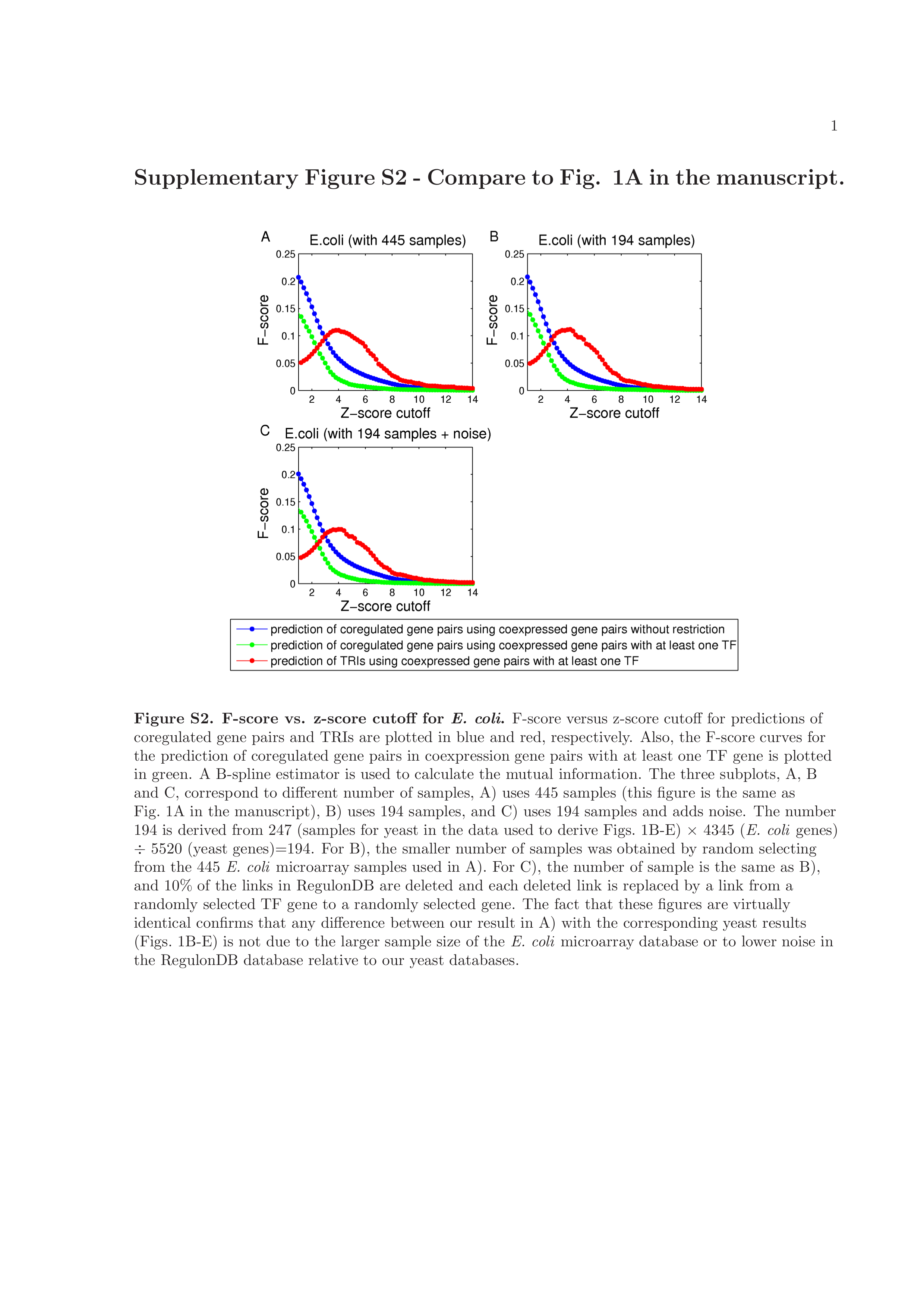

Supplement: Figure S2 — F-score vs. z-score cutoff for E. coli . F-score versus z-score cutoff for prediction of coregulated gene pairs and TRIs are plotted in blue and red, respectively. Also, the F-score curves for the prediction of coregulated gene pairs in coexpression gene pairs with at least one TF gene is plotted in green. A B-spline estimator is used to calculate the mutual information. The three subplots, A, B and C, correspond to different number of samples, A) uses 445 samples (this figure is the same as Fig. 1A in the manuscript), B) uses 194 samples, and C) uses 194 samples and adds noise. The number 194 is derived from 247 (samples for yeast in the data used to derive Figs. 1B–E) 4345 (E. coli genes) 5520 (yeast genes) = 194. For B), the smaller number of samples was obtained by random selecting from the 445 E. coli microarray samples used in A). For C), the number of sample is the same as B), and of the links in RegulonDB are deleted and each deleted link is replaced by a link from a randomly selected TF gene to a randomly selected gene. The fact that these figures are virtually identical confirms that any difference between our result in A) with the corresponding yeast results (Figs. 1B–E) is not due to the larger sample size of the E. coli microarray database or to lower noise in the RegulonDB database relative to our yeast databases. (TIFF) [file pone.0031969.s002.tif]

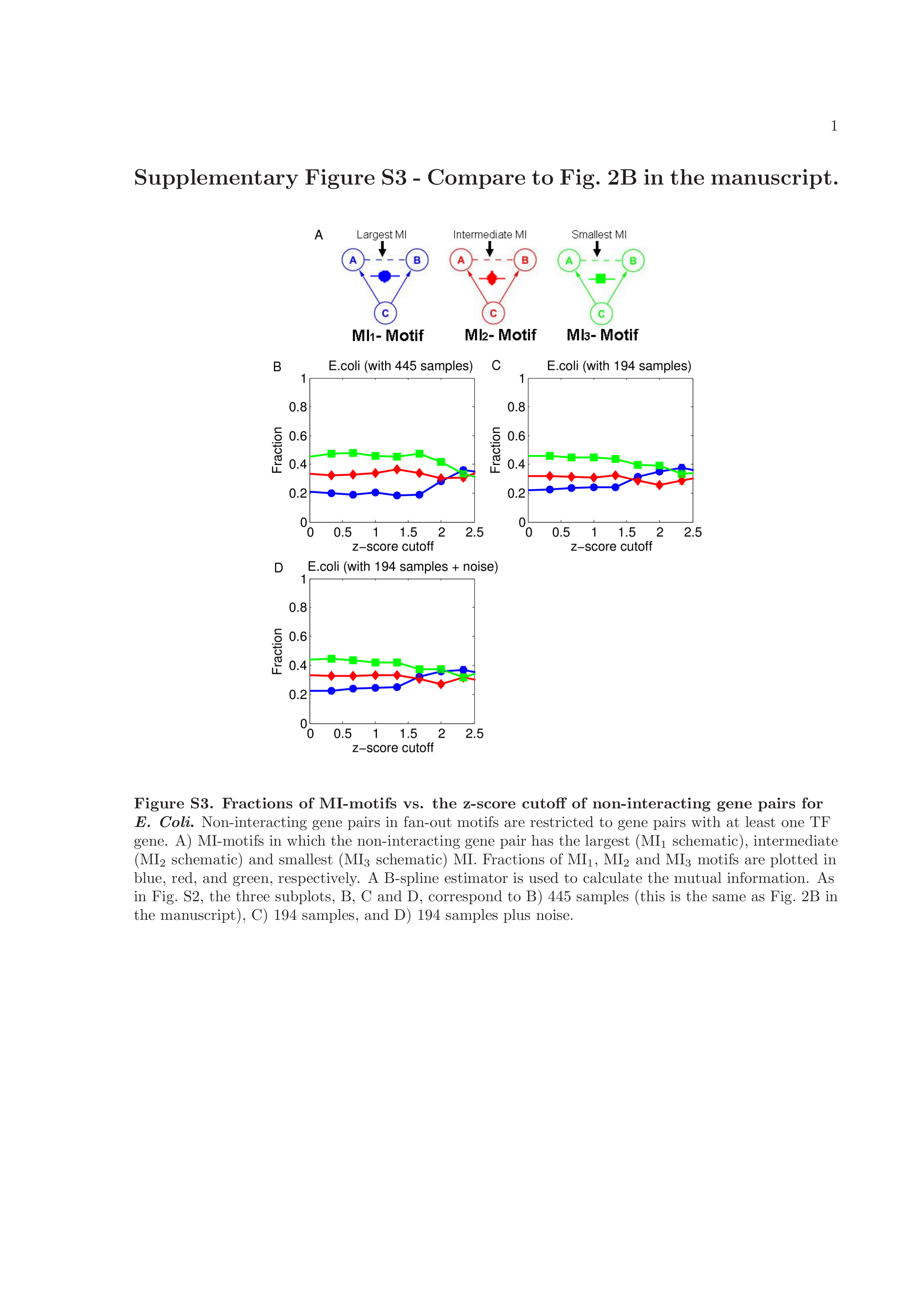

Supplement: Figure S3 — Fractions of MI-motifs vs. the z-score cutoff of non-interacting gene pairs for E. coli . Non-interacting gene pairs in fan-out motifs are restricted to gene pairs with at least one TF gene. A) MI-motifs in which the non-interacting gene pair has the largest (MI schematic), intermediate (MI schematic) and smallest (MI schematic) MI. Fractions of MI, MI and MI motifs are plotted in blue, red, and green, respectively. A B-spline estimator is used to calculate the mutual information. As in Fig. S2, the three subplots, B, C and D, correspond to B) 445 samples (this is the same as Fig. 2B in the manuscript), C) 194 samples, and D) 194 samples plus noise. (TIFF) [file pone.0031969.s003.tif]

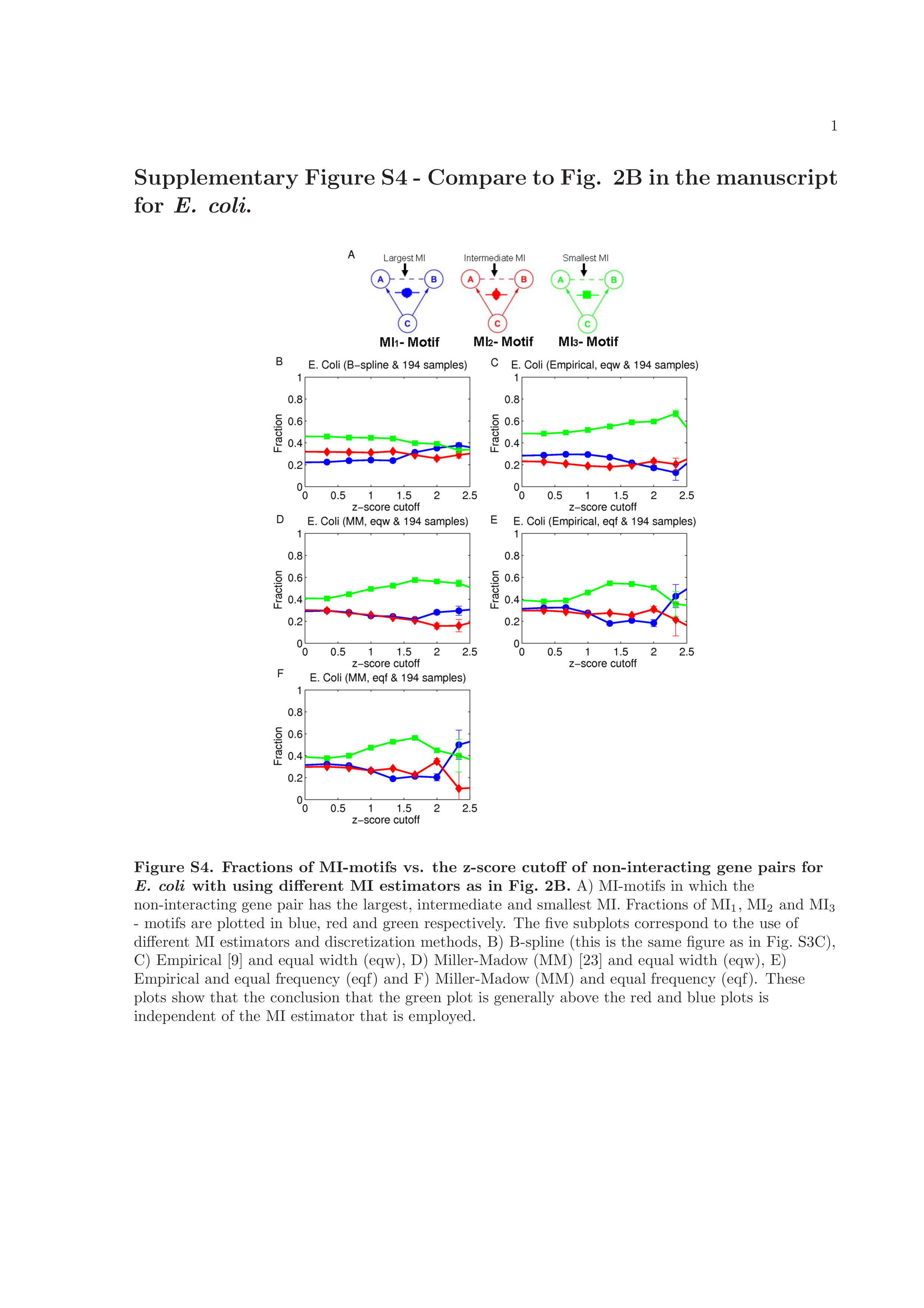

Supplement: Figure S4 — Fractions of MI-motifs vs. the z-score cutoff of non-interacting gene pairs for E. coli with using different MI estimators as in Fig. 2B . A) MI-motifs in which the non-interacting gene pair has the largest, intermediate and smallest MI. Fractions of MI, MI and MI - motifs are plotted in blue, red and green respectively. The five subplots correspond to the use of different MI estimators and discretization methods, B) B-spline (this is the same figure as in Fig. S3C), C) Empirical [9] and equal width (eqw), D) Miller-Madow (MM) [23] and equal width (eqw), E) Empirical and equal frequency (eqf) and F) Miller-Madow (MM) and equal frequency (eqf). These plots show that the conclusion that the green plot is generally above the red and blue plots is independent of the MI estimator that is employed. (TIFF) [file pone.0031969.s004.tif]

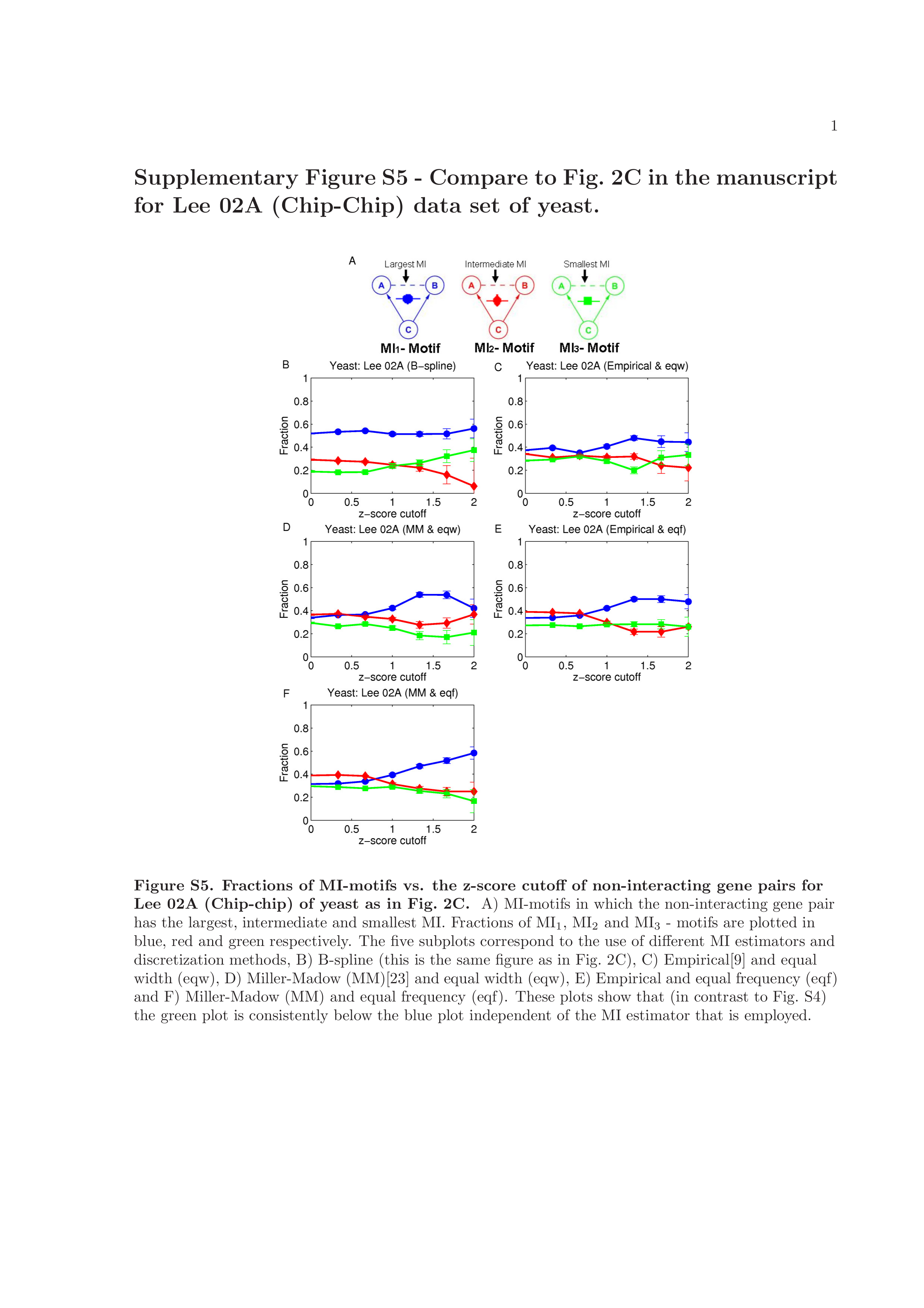

Supplement: Figure S5 — Fractions of MI-motifs vs. the z-score cutoff of non-interacting gene pairs for Lee 02A (Chip-chip) of yeast as in Fig. 2C . A) MI-motifs in which the non-interacting gene pair has the largest, intermediate and smallest MI. Fractions of MI, MI and MI - motifs are plotted in blue, red and green respectively. The five subplots correspond to the use of different MI estimators and discretization methods, B) B-spline (this is the same figure as in Fig. 2C), C) Empirical [9] and equal width (eqw), D) Miller-Madow (MM) [23] and equal width (eqw), E) Empirical and equal frequency (eqf) and F) Miller-Madow (MM) and equal frequency (eqf). These plots show that (in contrast to Fig. S4) the green plot is consistently below the blue plot independent of the MI estimator that is employed. (TIFF) [file pone.0031969.s005.tif]

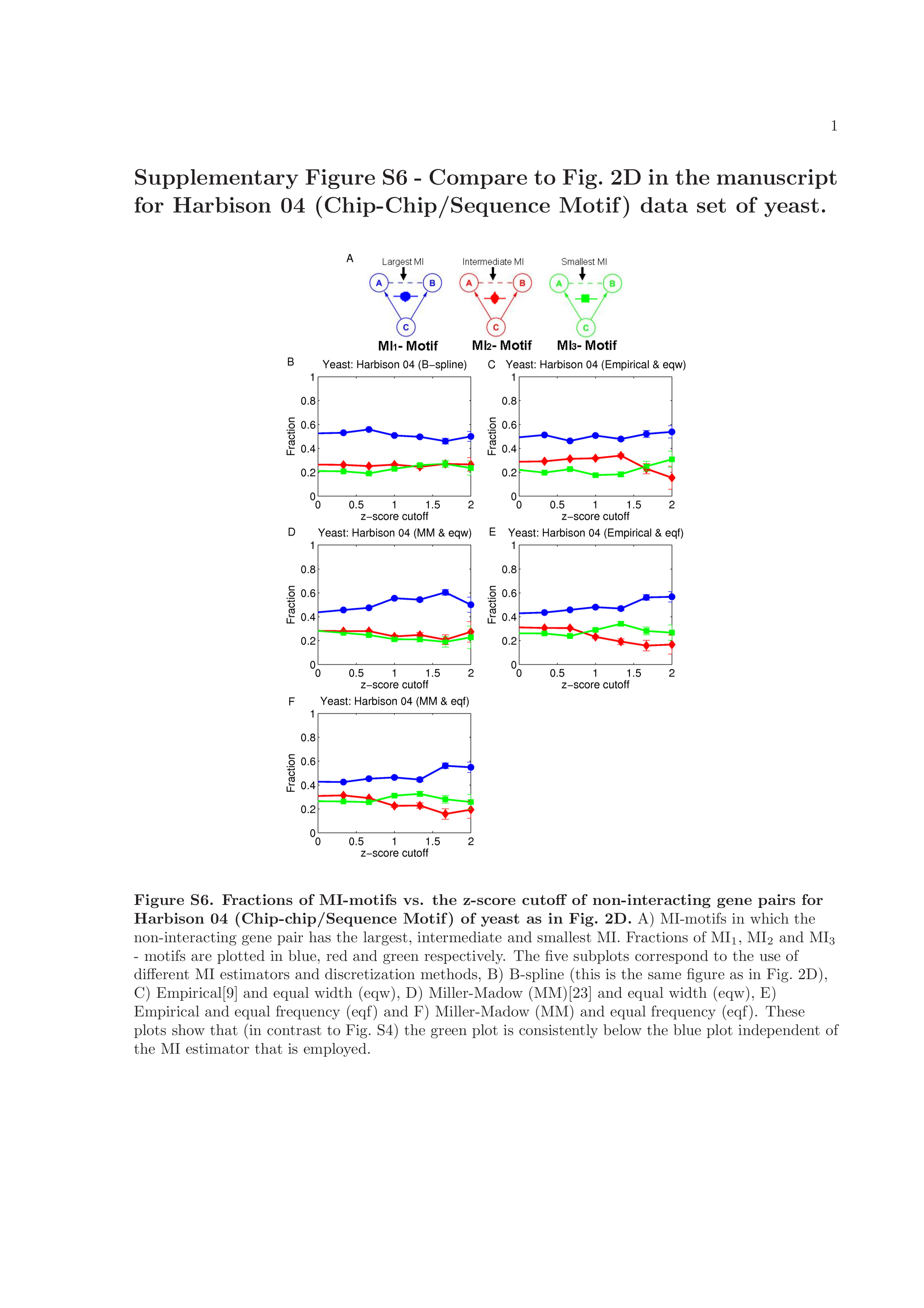

Supplement: Figure S6 — Fractions of MI-motifs vs. the z-score cutoff of non-interacting gene pairs for Harbison 04 (Chip-chip/Sequence Motif) of yeast as in Fig. 2D . A) MI-motifs in which the non-interacting gene pair has the largest, intermediate and smallest MI. Fractions of MI, MI and MI - motifs are plotted in blue, red and green respectively. The five subplots correspond to the use of different MI estimators and discretization methods, B) B-spline (this is the same figure as in Fig. 2D), C) Empirical [9] and equal width (eqw), D) Miller-Madow (MM) [23] and equal width (eqw), E) Empirical and equal frequency (eqf) and F) Miller-Madow (MM) and equal frequency (eqf). These plots show that (in contrast to Fig. S4) the green plot is consistently below the blue plot independent of the MI estimator that is employed. (TIFF) [file pone.0031969.s006.tif]

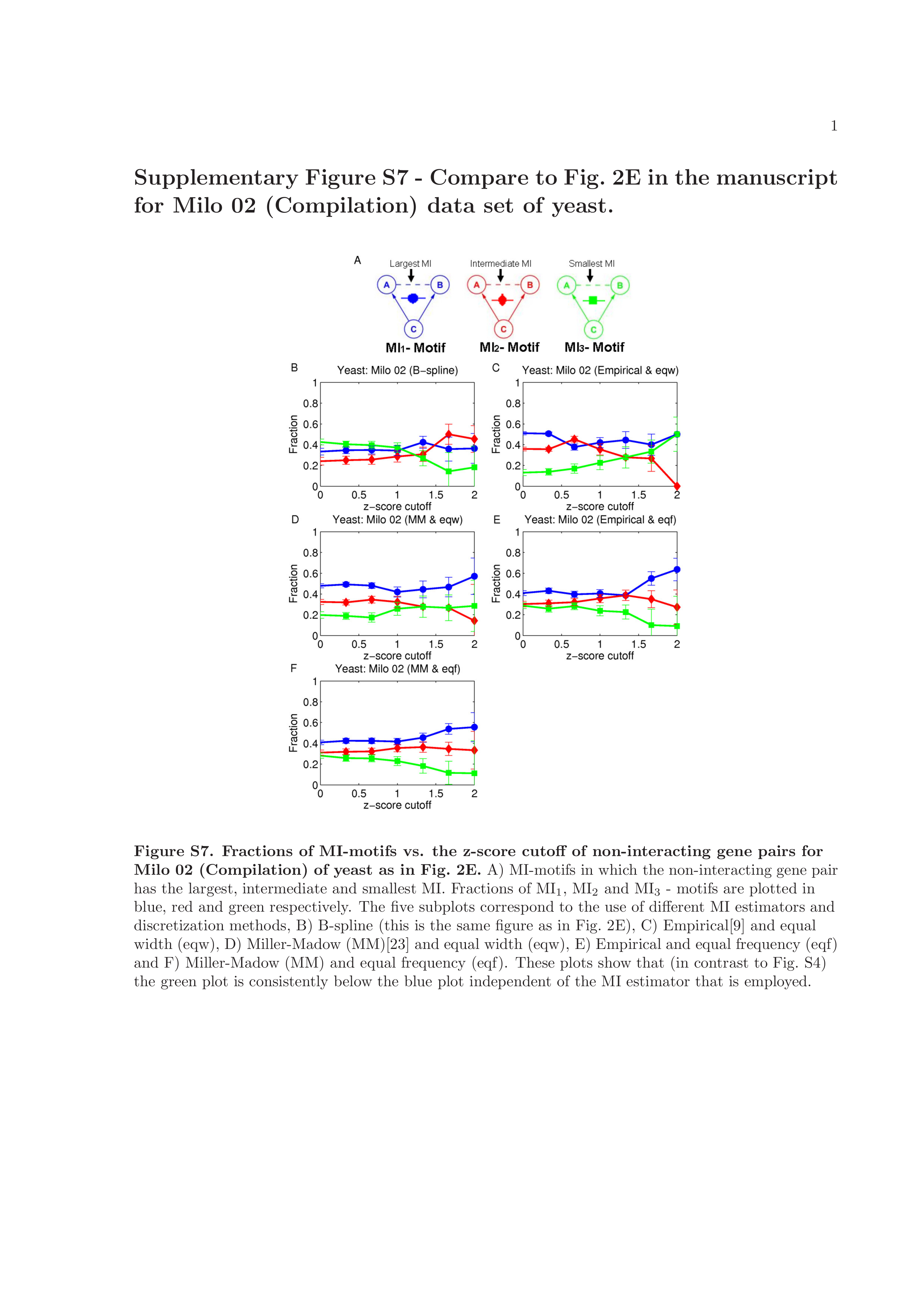

Supplement: Figure S7 — Fractions of MI-motifs vs. the z-score cutoff of non-interacting gene pairs for Milo 02 (Compilation) of yeast as in Fig. 2E . A) MI-motifs in which the non-interacting gene pair has the largest, intermediate and smallest MI. Fractions of MI, MI and MI - motifs are plotted in blue, red and green respectively. The five subplots correspond to the use of different MI estimators and discretization methods, B) B-spline (this is the same figure as in Fig. 2E), C) Empirical [9] and equal width (eqw), D) Miller-Madow (MM) [23] and equal width (eqw), E) Empirical and equal frequency (eqf) and F) Miller-Madow (MM) and equal frequency (eqf). These plots show that (in contrast to Fig. S4) the green plot is consistently below the blue plot independent of the MI estimator that is employed. (TIFF) [file pone.0031969.s007.tif]

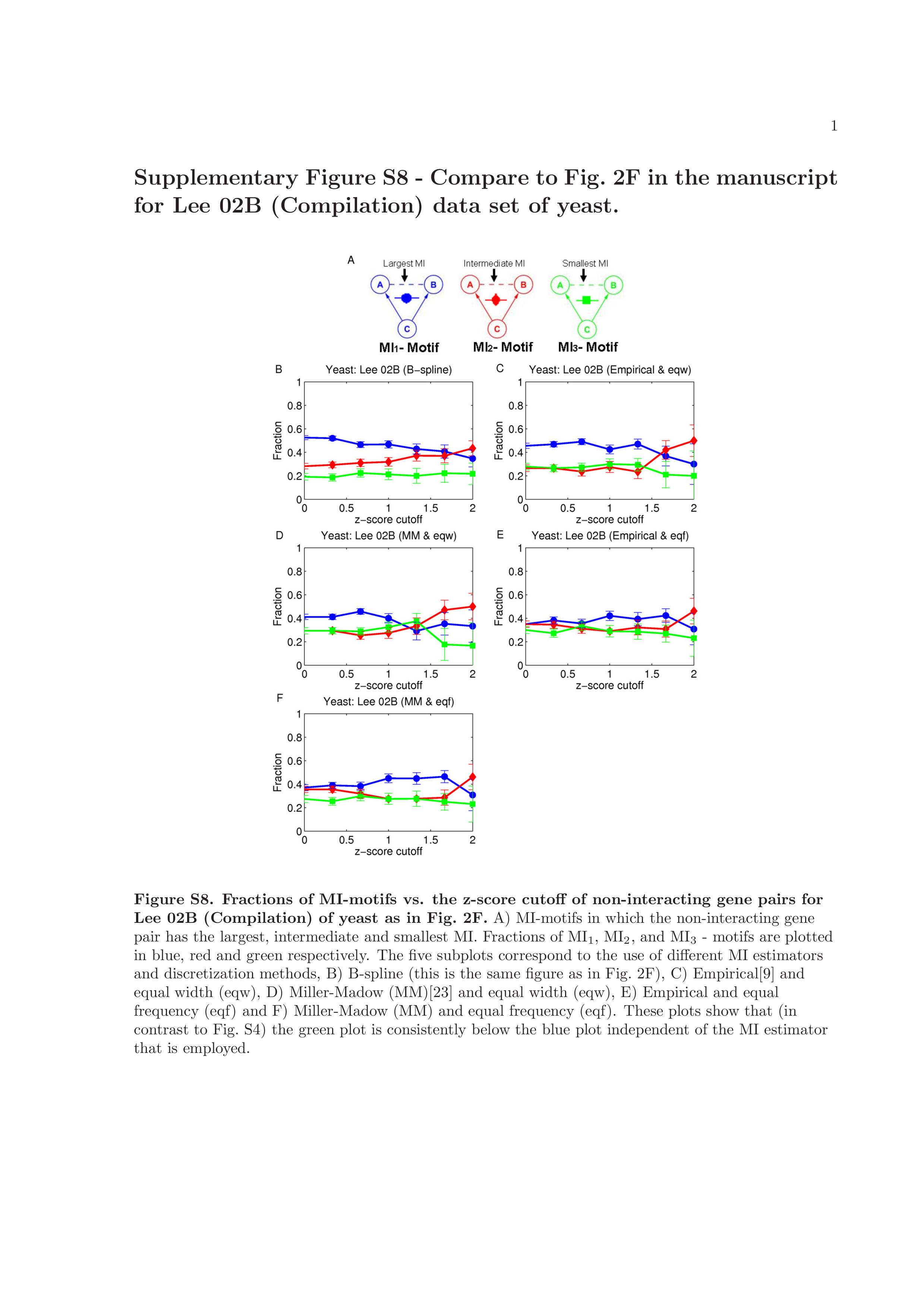

Supplement: Figure S8 — Fractions of MI-motifs vs. the z-score cutoff of non-interacting gene pairs for Lee 02B (Compilation) of yeast as in Fig. 2F . A) MI-motifs in which the non-interacting gene pair has the largest, intermediate and smallest MI. Fractions of MI, MI, and MI - motifs are plotted in blue, red and green respectively. The five subplots correspond to the use of different MI estimators and discretization methods, B) B-spline (this is the same figure as in Fig. 2F), C) Empirical [9] and equal width (eqw), D) Miller-Madow (MM) [23] and equal width (eqw), E) Empirical and equal frequency (eqf) and F) Miller-Madow (MM) and equal frequency (eqf). These plots show that (in contrast to Fig. S4) the green plot is consistently below the blue plot independent of the MI estimator that is employed. (TIFF) [file pone.0031969.s008.tif]
